# Sexual Interventions in the Metaverse: Attitudes Towards Novel Therapeutic Approaches, a Qualitative Study

**DOI:** 10.1111/hex.70004

**Published:** 2024-08-29

**Authors:** Ariana Vila, Rosa Romero‐Moreno, Celia Nogales‐Gonzalez, Andrew J. Ritchey, Juan Ardoy‐Cuadros

**Affiliations:** ^1^ Psychology Department, Faculty of Health Sciences Rey Juan Carlos University Madrid Spain; ^2^ Department of Sociology and Criminology Pennsylvania State University University Park Pennsylvania USA

**Keywords:** attitudes, avatar therapy, metaverse, qualitative study, sexual disorders, Telehealth, virtual reality

## Abstract

**Background and Objective:**

Mental health treatment for psychosexual problems is effective, but treatment rates are low. Metaverse‐based therapy offers one solution to increase overall treatment rates. Understanding attitudes towards this novel approach could lead to wider adoption of metaverse‐based therapy, resulting in higher treatment rates for psychosexual problems.

**Methods:**

Twenty‐one participants across three focus groups of different ages shared their perceptions and attitudes about metaverse‐based therapy broadly and for treating sexual disorders. A content analysis of the transcribed text from the focus groups using qualitative data analysis software was conducted.

**Results:**

Participants identified several perceived benefits of metaverse‐based intervention, including avoiding the perceived embarrassment of going to a clinic and accessing patients (a) with diverse physical or mental functionality, (b) living in remote areas and/or (c) balancing different family/work obligations or duties. The two main concerns with metaverse‐based therapy were the fear of online therapy being less personal than traditional therapy and the technological fluency needed. Clarifying their acceptance of the therapy, participants reported that they would be more likely to engage in metaverse‐based therapy if they trusted their therapist. Also, although it might be effective for mild and moderate disorders, participants were more reluctant about its use for severe mental illness.

**Conclusions:**

Results suggest that attitudes towards metaverse‐based intervention are mainly positive, since it removes some barriers that hinder access to psychological treatment in general and, specifically, for problems of a sexual nature.

**Patient and Public Contribution:**

During the design stage, a person with sexual difficulties was consulted to understand the patient's perspective. Members of the public advised the implementation of the focus groups. Three potential service users were involved in the coding of the text during the content analysis.

## 1 Introduction

Sexual dysfunctions present problems for women and men worldwide [1, 2]. The prevalence of sexual dysfunctions is estimated to affect between 30% and 70% of the population [3, 4, 5, 6, 7]. Given the levels of distress related to these conditions [4], sexual dysfunctions could be considered a serious health problem. Considering that psychosexual treatments have been proven to be effective in treating sexual disorders [8, 9, 10, 11], it is important to analyze the motives of individuals who do not seek psychological help. Previous literature reveals that there are no rigorous studies regarding the median rates of untreated sexual dysfunctions as there are for other disorders such as depression [12]. However, it is known that people rarely request psychological assistance in such cases [13, 14]. Reasons for not seeking help to treat sexual dysfunctions (i.e., barriers to sexual treatment) include unrealistic expectations about treatment, intrinsic motivation to change [13], shame, guilt [14], lack of time and limited privacy during consultations [15]. There is a significant gap between providers' perceptions and patients' requirements regarding discussions related to sexual health [16]. A qualitative study undertaken with women [15] identified that participants tended to avoid discussing their sexual concerns with general practitioners due to personal discomfort. Additionally, shame and attitudes towards later life sexuality prevent older people from discussing sexual problems [17, 18]. Regarding facilitators, information provision seems to be an important factor in help‐seeking [19]. Given the difficulties in getting help for sexual problems, it appears doubtful that the traditional ways of providing mental healthcare alone will be able to meet the demands. That said, e‐mental health interventions may offer several advantages to addressing service demands effectively.

E‐mental health has no agreed field‐specific definition. It is generally considered to be the group of treatments, mental health services and information that have been adapted for delivery via the internet or related technologies [20, 21]. They include not only initiatives delivered directly to mental health service users [22, 23] but also mental health promotion and prevention [24, 25], screening [26] and staff training [27]. Attitudes towards e‐mental health interventions seem to be mainly positive [28, 29, 30, 31, 32, 33]. E‐mental health interventions have been proposed to improve sexuality [34], including treating female orgasmic disorder [35], sexual interest/arousal disorder [36] and sexual aversion [37].

The e‐mental health intervention focused on for the current study is metaverse therapy or metaverse‐based therapy. The term metaverse describes a collective, virtual, shared space that is typically accessed through the internet. It is a three‐dimensional digital world that is designed to simulate a physical world but includes various elements that are not present in real life [38]. The popularity of metaverses is on the rise, and they can be used to treat sexual disorders [39]. It is anticipated that metaverses will have a significant impact on the future of the internet and technology. Metaverse therapy consists of exploring issues through a virtual representation of the patient (i.e., avatars) in a virtual environment. In addition to the benefits common for all e‐health (e.g., easy access to treatment, availability and flexibility) [39], metaverse therapy allows other specific benefits, such as the possibility to encourage the externalization of experiences, thoughts and feelings [40, 41], the opportunity to gain insight and develop empathy [42] and the option to avoid the embarrassment of going to a clinic [39]. Metaverse therapy might help users with sexual issues get the help that they need in the following ways: first, metaverses have the capacity to overcome geographical barriers and provide remote accessibility [25, 39]. Second, they offer enhanced flexibility in scheduling and availability [39]. Finally, they can offer personalized and tailored experiences through interactive digital platforms, incorporating features like self‐assessment tools and educational resources.

Attitudes towards the use of metaverses to receive psychological interventions have been understudied, probably because of the novelty of the topic. A screening of the literature regarding attitudes towards metaverse therapy revealed only three studies [40, 41, 42], and none of them was focused on sexual dysfunctions or analysed the facilitators and barriers to this new form of treatment. Given this dearth of knowledge, this study specifically aims at analysing individuals' attitudes about using the metaverse to treat mental and sexual disorders. Studying these attitudes could contribute towards understanding, adapting and improving sexual interventions, thus identifying new approaches to provide treatment to people who currently are not seeking help. Additionally, it would reveal facilitators and barriers to treatment and suggests ways to encourage the facilitators and overcome the barriers.

## 2 Method

In the context of the scant existing literature on the research topic, a qualitative methodology was decided to be the best preliminary approach to allow a wide exploration of the topic.

The specific methodological strategy was based on combining focus groups and discourse analysis. Focus groups are a technique especially suited for exploratory purposes [43]. Three focus groups—divided by age to help identify any age‐based differences—were carried out to assess people's perceptions and attitudes about (1) metaverse‐based therapy for mental health interventions (any disorder) and (2) metaverse‐based therapy for treating sexual disorders. Previous literature stated that more than 80% of all themes were discoverable within two to three focus groups [44]. On the basis of this, it was decided to conduct three focus groups, with additional focus groups being possible if the information overload goal was not achieved.

A content analysis was conducted after these focus groups to identify relevant points in participants' experiences [45]. The analysis was guided by the health belief model (HBM) [46], the technology acceptance model (TAM) [47] and previous research on attitudes towards e‐mental health interventions (e.g., they are perceived as more acceptable for less serious disorders or when patients have had previous e‐mental health experience [28, 29, 30], and seen as less acceptable when they are used as stand‐alone interventions [34, 35]). The focus group protocol, along with this study more broadly, was approved by the Ethics Committee of the Rey Juan Carlos University (Spain) (Registration Number: 0604202109921).

### 2.1 Participants

Participants were 21 volunteers recruited through an invitation to participate that was posted within Rey Juan Carlos University and distributed online through social media and personal communication applications. Groups were divided by age—aged 18–39 years (young people; *n* = 7), 40–65 years (middle‐aged; *n* = 6) and 66–81 years (older people; *n* = 8). Inclusion criteria were (a) being at least 18 years old, (b) not having met or had any previous contact with other participants (separate interviews with participants were conducted to gather information about their social networks), (c) not having met or had any previous contact with the facilitator and (d) being non‐experts in the research topic. The exclusion criterion was having any difficulty (e.g., cognitive impairment) that could have restrained the correct comprehension of the study's characteristics.

Mixed gender groups, with different cultural backgrounds and socioeconomic statuses, were used to ensure a more comprehensive representation of the population of interest. Including a diversity of perspectives allowed us to avoid bias and stereotyping, adding to validity and generalizability and resulting in richer discussion and interaction [43, 48, 49, 50] (i.e., participants had unique experiences, opinions and communication styles, which fostered a more stimulating and productive exchange of ideas). Table 1 depicts participants' demographic information and previous experience with psychology treatments, separated by focus group. These data were collected during the engagement phase through open‐ended questions. The questions were moderated by the first author.

**Table 1:** Demographic information and previous psychological treatments.

	Group 1	Group 2	Group 3
Age range	18–39	40–65	66–81
Mean age (standard deviation)	27 (SD = 5.89)	53.5 (SD = 8.81)	75.38 (SD = 3.97)
Number of participants (*N* = 21)	7	6	8
Male	3	3	4
Female	4	3	4
Socioeconomic status			
High	2	3	2
Medium	1	2	4
Low	4	1	2
Ethnicity			
White	3	5	3
Asian	1	0	1
Black	1	0	2
Hispanic	2	1	2
Place of residence			
Urban	3	4	4
Rural	4	2	4
Education			
Undergraduate	1	2	2
Professional training	2	1	2
Graduate	1	2	3
Postgraduate	3	1	1
% Previous psychology treatment	42.86%	33.33%	12.5%
Was it a good experience?	66.67%	100%	100%
% Everyday internet use	100%	100%	62.5%

Overall, focus groups were diverse in similar ways, allowing for more confident comparison between groups. The middle‐aged focus group tended to be more White than other groups. Also, the older age focus group used the internet less and had less experience with prior psychological treatment—it seems likely that these characteristics are representative of the older population in general when compared to younger groups. That said, it also complicates whether potential differences between the older age group and younger age groups could be due to age, previous experience with psychological treatment or internet use.

### 2.2 Procedure

Twenty‐one participants aged 25–81 years were divided by age into three focus group sessions. Each session lasted approximately 90 min. All sessions were facilitated by the first author, using focus group guidelines found in the literature [43, 48, 49, 50]. The following guidelines included instructions about group composition (i.e., selection and arrangement of participants on the basis of specific criteria relevant to the research objectives), the moderator's role (e.g., guiding the discussion, ensuring that all participants have an opportunity to express their opinions), content (i.e., keeping the conversation focused on the topic) and duration of the session.

Participants viewed two videos created for the focus groups so that they could get familiarized with the research topic. Given the novelty of the topic, it was important to guarantee that participants understood the context, key concepts (e.g., what a metaverse is, how it works and what it is commonly used for) and relevant issues (e.g., how psychological treatment would take place) before engaging in the discussion. The videos helped (a) ensure that participants started with a similar level of knowledge and understanding about the topic and (b) facilitate engagement and accessibility (when compared to reading material). The videos included (1) an example of how traditional therapy and metaverse‐based therapy would work for several disorders, including sexual issues, and (2) a virtual tour through the metaverse Second Life. Second Life is one of the most well‐known, currently active metaverses. Second Life was selected from other available metaverses because it is one of the most well‐known, currently active metaverses, and it includes specific characteristics that make it suitable for conducting psychological therapy (e.g., the ability to use virtual objects while conducting therapy that may be expensive or difficult to obtain in real life, and the ease of building specific environments without having technical expertise). After watching the two videos, the participants signed the informed consent and were presented with three main types of questions in the focus groups (the script can be found as File S1):

1. Engagement questions: This category of the focus group protocol included questions about areas of interest for the study, not directly related to perceptions and attitudes about metaverse‐based therapy. These questions introduced participants to the topic. Some examples were if they had gone to traditional therapy before or if they used internet daily (e.g., Have you ever gone to therapy before?).
2. Exploratory questions: The general content of the focus groups' discussions focused on participants' attitudes, so the protocol included questions that revolved around three main categories: (1) advantages of using a metaverse‐based therapy for mental health interventions/treating sexual disorders (e.g., What advantages do you think this kind of therapy may have over traditional treatments?), (2) treatment confidence/trust (strengths and weaknesses, e.g., Would you use it?) and (3) problems/barriers that they see (e.g., Is there any reason why you would reject a metaverse‐based therapy?).
3. Exit questions: These questions served the purpose of exploring anything missed during the discussion (e.g., Is there anything else you would like to share or say?).

### 2.3 Analysis of Responses

After the focus groups were conducted, the audiotapes were transcribed. A content analysis of these transcriptions was performed using the program ATLAS.ti 9 to codify the text. One researcher and three potential service users, all trained in qualitative research, independently coded the content from the focus groups through an open‐coding process. They systematically read through the data and assigned descriptive codes to segments of text that represented meaningful concepts, ideas or themes. The codes could have been short phrases or labels that captured the essence of the content. Codes were based on HBM (which includes six constructs: susceptibility, severity, perceived benefits, perceived barriers and cues to action) and TAM (which includes four constructs: perceived usefulness, perceived ease of use, intention to use and facilitating conditions) models. Inductive coding was used to derive new themes or categories from the data [51, 52] instead of using pre‐existing ones.

After the coders familiarized themselves with the data (i.e., reviewed interview transcripts to gain a comprehensive understanding of the content and context), they independently coded the transcribed audiotapes. Next, they met to compare the codes and created a code list (i.e., a list of codes based on the concepts and themes identified in the data). This list was then applied to the relevant segments of data to verify their relevance and to refine and adjust the final code list. A final 100% agreement in the coding process was reached, and the codes were then grouped together on the basis of their similarities or connections, forming broader themes or categories. At the end of the process, the categories and subcategories below were retrieved and classified according to whether they were acting as facilitators or barriers.

## 3 Results

Participants' characteristics, including demographic information and previous experience with psychological treatments, are depicted in Table 1.

The results of the analysis revealed a total of four categories (new themes derived from the data during analysis) and nine subcategories (Table 2). There are seven subthemes acting as facilitators: (1) Using it as a tool to avoid the embarrassment/discomfort of having to go to a clinic; (2) gaining access to populations with diverse physical or mental functionality; (3) gaining access to populations living in rural areas, where it may be more difficult to access psychological treatments; (4) being an effective treatment for sexual disorders (i.e., being a reliable modality of treatment); (5) helping with time scheduling around other, family/work obligations; (6) having particular treatment advantages derived from platform use; and (7) being a useful therapeutic tool, when used as part of a mixed intervention (i.e., in person and inside a metaverse). In addition, there are two subthemes acting as barriers, related to concerns associated with using a metaverse‐based therapy for the treatment of sexual/mental disorders: (1) being less personal and (2) being only for young people.

**Table 2:** Themes and subthemes from the focus groups.

Theme/subtheme	Definition/content	Example	Facilitator or barrier
Embarrassment	Feeling of discomfort, self‐consciousness or shame related to seeking mental health treatment.		
Avoid embarrassment/discomfort	Participant feels embarrassed about sexual/psychological therapy or indicates being embarrassed as a barrier to receive treatment.	‘I feel maybe that (metaverse‐based therapy) would help […] It/would be easier if I don't have to go to the clinic, I feel safer at home’ (L., female, 26 years old). ‘[…] this is something (sexual therapy) I won't do. I feel so embarrassed of thinking about it’ (J., male, 45 years old). ‘Oh no, please! How would I say that [sexual difficulties] to my doctor? Can you imagine, a woman my age!?’ (M. C., female, 66 years old).	Facilitator
Accessibility	Ease with which individuals can obtain and utilize mental health services.		
Access to population with specific characteristics	Participant mentions that persons with diverse physical and/or mental functionality could benefit from an online intervention.	‘Even for adolescents that maybe have it more difficult to treat sexual matters as they live with their parents and asking for that kind of treatment maybe [the adolescents] wouldn't be comfortable sharing with [their parents]’ (F., male, 23 years old). ‘I'm lucky because I can walk, and drive around, and travel, and take the subway … but I imagine not everyone has my capacities […] maybe for them it is a very good option’ (M., female, 52 years old). ‘[…] also, for disabled people, I think they would benefit’ (M., female, 73 years old).	Facilitator
Access to population living in rural areas	Participant mentions that persons who live in remote and/or rural areas could benefit from an online intervention.	‘Psychological issues are frowned on […] this seems like a game, and I think it will help in this way’ (J. C., male, 31 years old). ‘In the countryside people are narrow‐minded, I lived there for half my life, and I wouldn't have dared go to a clinic’ (M., female, 52 years old). ‘If I'd had access to this when I was young and living in the village, it would've been a different story’ (E., female, 78 years old).	Facilitator
Time schedule and family/work conciliation	Participant indicates that a metaverse‐based therapy helps balance different obligations or duties.	‘I don't have time for therapy […] this (treatment) saves time’ (R., female, 26 years old). ‘It isn't only the hour you spend there, [it] is going and coming back […] traffic is horrible and taking the metro is even a worse option’ (J., male, 45 years old). ‘With kids under three, therapy seems impossible, but if I can do it from home maybe they can watch TV while I'm there’ (M., male, 40 years old).	Facilitator
Utility	The ability to produce positive outcomes and alleviate mental health symptoms or distress.		
An effective treatment for sexual disorders	Participant views the treatment as a good option for sexual and/or mental disorders.	‘[…] this (treatment) is way better than traditional ones’ (J. A., male, 18 years old). ‘I don't know how this sexual therapy works, but I think it (metaverse‐based treatment) sounds interesting for practicing’ (U., female, 56 years old). ‘I imagine the therapist could be with you while you learn […] this would help me, like I'm not alone’ (E., female, 78 years old).	
Particular treatment advantages derived from platform use	Participant indicates advantages derived from using the platform (e.g., entertaining, pleasant or handy).	‘[…] sounds entertaining’ (R., female, 26 years old). ‘It's the opposite of boring’ (C., male, 29 years old). ‘It's really accessible, you can always use it’ (E., male, 25 years old).	
A useful therapeutic tool	Participant views the treatment as an effective therapeutic tool, used for some specific tasks in conjunction with traditional treatment.	‘[…] I don't know, maybe as part of the treatment, combined with some sessions or other techniques, I think it'd be wonderful’ (J. C., male, 31 years old). ‘Why not? For sure if my therapist proposes using it, I'll say yes’ (J., male, 45 years old). ‘I'd like the combination of this and traditional therapy’ (M. C., female, 66 years old).	
Reservations	Obstacles or concerns when considering seeking metaverse‐based healthcare.		
Less personal	Participant expresses a fear of metaverse therapy being less personal.	‘I don't know if information will be lost, like all the body language part’ (A., male, 31 years old). ‘I'm not sure if this kind of online therapy [metaverse therapy] is like traditional therapy […] I think [it] is less personal’ (N., female, 49 years old). ‘I understand the benefits, but I'd prefer something more personal like in‐person treatments’ (M., female, 73 years old).	Barrier
Only for young people	Participant indicates age‐related issues.	‘This is great for future generations. My granddaughter was born with a telephone in her hands. I'm sure she'll love this’ (F. J., male, 73 years old). ‘I don't know if I will be comfortable with all the things you have to learn. Maybe [it] is too much’ (U., female, 54 years old). ‘If I must pay attention to what my avatar is doing along with what I'm saying, maybe I'll get lost. I need to try it, but that's what I think’ (E., female, 60 years old).	Barrier

### 3.1 Embarrassment

#### 3.1.1 Avoid Embarrassment/Discomfort

Through the group sessions, one consistent topic was ‘embarrassment’. For sexual disorders, along with other diseases, 100% of participants felt that going to a clinic was embarrassing. Thus, metaverse treatment may allow them to reach out for help that they otherwise would not seek. There was no difference by age or gender.

### 3.2 Accessibility

#### 3.2.1 Access to Population With Specific Characteristics

One hundred percent of the participants agreed that persons with diverse physical and/or mental functionality could benefit from an online intervention. In each group, this topic emerged in different forms, but they all came to terms with the idea.

#### 3.2.2 Access to Populations Living in Rural Areas

In every group session, participants discussed how a metaverse‐based intervention may ease treatment for those who live in remote areas, where people tend to have negative attitudes towards psychotherapy in general. One participant in each group mentioned the inconveniences of going to psychological therapy in their hometowns. All participants usually agreed that rural areas may benefit from metaverse therapy.

#### 3.2.3 Time Schedule and Family/Work Conciliation

Participants in the focus groups mostly agreed that a metaverse‐based therapy may allow patients to balance different obligations or duties (85.71%: one person in each group thought that their schedules were so busy that even a metaverse‐based treatment would not fit in their schedules). It also seemed like a perceived advantage for big cities.

### 3.3 Utility

#### 3.3.1 An Effective Treatment for Sexual Disorders

The groups reported metaverse‐based treatments as a good option for different sexual disorders. They agreed that for sexual‐related issues, they would prefer this kind of treatment over traditional in‐person therapy. Regarding mental disorders, they did not reach such an agreement; if having a mental issue, some of the participants would prefer to receive traditional in‐person therapy (57.14%), whereas others would choose metaverse therapy (42.86%).

#### 3.3.2 Particular Treatment Advantages Derived From Platform Use

Participants tended to label the content of a metaverse‐based treatment as entertaining (61.9%), pleasant (52.38%) or handy (57.14%). These characteristics were highlighted when compared to traditional treatment.

#### 3.3.3 A Useful Therapeutic Tool

Participants agreed (100%) that a metaverse‐based treatment would be an efficient therapeutic tool, used for some specific tasks during treatment. This category does not exclude previous attitudes; in this light, participants stated that metaverse may and may not be a replacement for traditional in‐person therapy.

### 3.4 Reservations

#### 3.4.1 Less Personal

The participants voiced their fear of metaverse therapy being less personal. Participants did not present this issue as a specific concern related to metaverse‐based treatments, but something that every online intervention (i.e., not in‐person therapies) shares. The individuals mostly agreed about the impersonal nature of therapy online, not only of metaverse‐based treatments but in general (66.66%).

#### 3.4.2 Only for Young People

Differences between groups were found regarding age‐related issues; younger people saw themselves as possible users (85.71%), whereas older participants would recommend metaverse‐based therapy to their children (87.5%). They debated if they would be capable of using it and, even though some agreed that they could learn, others discussed if maybe it was too much effort. Elderly individuals showed reticence about both metaverse and online interventions, but they showed higher reluctance towards therapy within a metaverse. One hundred percent of the participants perceived therapy in the metaverse to be more challenging to grasp. Older participants were not used to metaverses as they were to other forms of remote therapy technologies, such as videoconferencing or apps.

**Figure 1:**
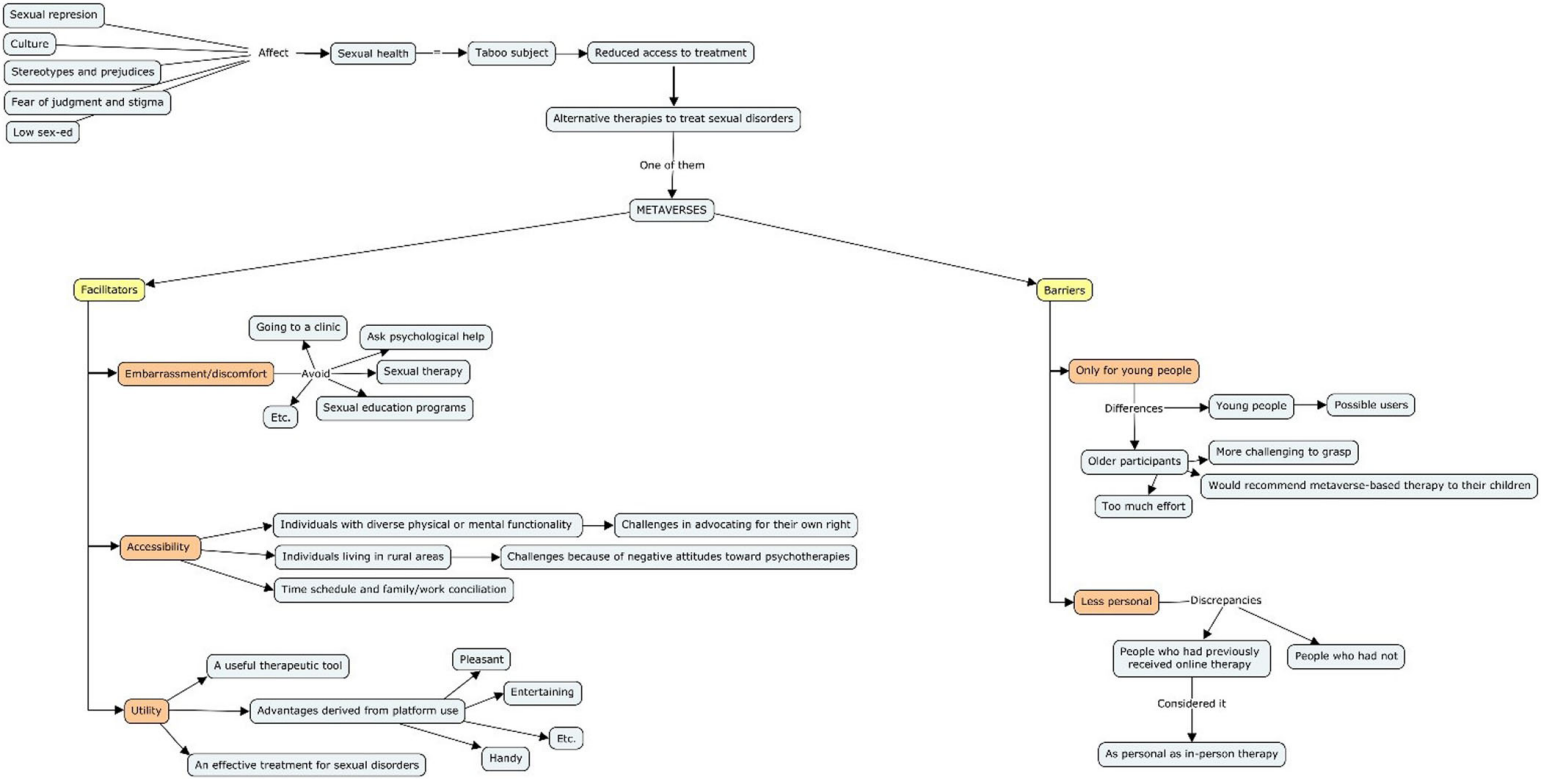
Information about categories' relationships.

In Figure 1, the categories, and connections or patterns, that emerge from the data (i.e., trends or recurring ideas within the data set) are displayed in schematic form.

Additionally, the analysis revealed other interesting data, such as: (1) There was no difference in attitudes towards metaverse‐based therapy between those who had received previous psychological treatment and those who did not. (2) Participants agreed that their decision to use the technology or not would depend on their trust in their therapist. (3) They also agreed that they trusted a metaverse‐based treatment for mild and moderate disorders but not for severe mental illness (e.g., ‘such as schizophrenia’). (4) There were differences in attitudes between those who had received previous online psychological treatment and those who did not; one person within the total sample who had previously received online therapy during the Covid lockdown, and her opinion differed from the rest: ‘I tried (online therapy) because I have no other options. We were in lockdown and, even [though] I had my reservations, it was the best thing I did. There was no difference with traditional therapy, I assure you’ (R., female, 26 years old).

Moreover, in the young people group, different scenarios were discussed, for example, a treatment for desire disorders in which the metaverse could be used to explore new forms of pleasure, or just using the virtual environment to try some sexual fantasies that they do not feel comfortable sharing with their real‐life partner. The older people group agreed with this last topic and discussed (1) how imposed sexual repression led them to avoid specific sexual behaviors and (2) how the possibility to explore ‘forbidden’ sexual areas would have helped them have a healthier relationship with their sexuality.

## 4 Discussion

Using focus groups, this study has the goal of exploring the general public's attitudes towards a metaverse‐based intervention for treating mental health and sexual disorders to describe barriers and facilitators to seeking this new form of intervention. A content analysis revealed attitudes centred around four themes (categories) and nine subthemes. The connections between these themes provide a tentative explanation of the barriers that prevent people with sexual issues from receiving the psychological help they need, as well as why alternative therapies may present facilitators that offer a possible avenue towards treatment. Overall, the results of the study found that participants viewed metaverse‐based interventions as positive, though there were still some concerns. Below, the implications of each of the nine subthemes are discussed, and further reflection on the study's limitations is provided.

To begin, focus groups identified the reduction of embarrassment as one possible facilitator for treatment. Participants viewed metaverses positively as a way to avoid the feeling of embarrassment associated with visiting a clinic. Embarrassment may be particularly possible for sensitive issues where individuals may feel stigmatized or ashamed, such as with sexual disorders. To our knowledge, attitudes regarding these perceptions of embarrassment have not been reported in previous literature. What is known is that sexuality is still a taboo subject [13, 14]. People feel ashamed about having to openly recognize that they have a sexual problem, so they avoid looking for psychotherapeutic help. This reluctance to seek psychological support can exacerbate sexual issues, leading to a decline in overall well‐being. E‐mental health—not just metaverse therapy—provides a discreet option for individuals to seek help without the embarrassment of physically visiting a health centre. It should be noted that the study participants' perception of clinics or health facilities as embarrassing also indicates the need for healthcare providers to create a safe and comfortable physical environment for patients to seek psychological help.

The next three facilitators revealed by the focus groups concern access to healthcare. First, participants reported that a metaverse‐based intervention may help persons with diverse physical or mental functionality to overcome the inconveniences of trying to get traditional therapy. By being a more accessible and inclusive option, metaverse interventions could help reduce disparities in access to care [53]. Healthcare providers should consider e‐mental health as a way to ensure inclusive healthcare for individuals with physical disabilities or mental health conditions. Results suggest that e‐mental health grants individuals access to the same quality of care, regardless of disabilities [53]. The second attitude about access highlighted in the focus groups concerned geographical barriers to healthcare. Focus groups discussed that individuals living in remote areas may face challenges accessing psychological therapy due to the long distances to physical clinics and the limited resources (e.g., time and money) to reach those clinics. Beyond physical barriers, cultural barriers may also be present. For example, the participants observed that people in rural areas tend to have negative attitudes towards psychotherapies in general. These negative attitudes may contribute to a reluctance to seek psychological therapy in rural areas [54]. Although cultural reluctance would still need to be addressed, e‐mental health interventions eliminate physical barriers and increase access to healthcare, offering greater access to individuals in rural areas. Finally, participants considered metaverse‐based therapies to increase access to healthcare by offering greater flexibility in scheduling, helping balance therapy with other time obligations. A metaverse‐based treatment was viewed as overcoming usual barriers to in‐person treatment, including full‐time work, childcare responsibilities, urban traffic congestion and long travel times. Furthermore, participants reported that e‐mental healthcare expands mental health access to everyone who may find it difficult to schedule appointments during traditional business hours. By offering e‐mental health interventions, healthcare providers can facilitate access to treatment to patients with busy schedules.

Among the benefits of using e‐mental health in general, the attitudes around access summarized above are congruent with those identified in previous literature: being easy to access treatment (for hard‐to‐reach populations, persons who feel stigma and young individuals), availability, reduced costs and flexibility [35, 55, 56]. Beyond these attitudes about the general benefits of e‐mental health, focus groups revealed attitudes about the specific benefits and utility of metaverse‐based therapy.

Encouragingly, focus groups revealed three positive attitudes regarding the utility or effectiveness of metaverse‐based therapy. These three attitudes are specifically in line with the TAM [47] model as a framework for understanding attitudes towards technology usage behaviors. The perceived usefulness and ease of use, along with the facilitating conditions described above (e.g., easy‐to‐access treatment, availability, reduced costs, flexibility), may offer an explanation for why there are a much greater number of facilitators than barriers to treatment. Regarding the first two attitudes, participants considered metaverse‐based therapy as a good option (a) for the standalone treatment of different sexual disorders and (b) as a therapeutic tool used in tandem with other treatment modalities. It is important to highlight that these are the subjective perceptions of non‐experts, but these results show that lay people are open to using metaverse therapy to address sexual disorders. Specific tasks in the metaverse, such as exposure therapy or skill‐building exercises, could complement traditional therapy and improve treatment outcomes [57]. This could help address the unique needs and challenges of individuals with sexual disorders.

Besides using the metaverse as an intentional therapeutic tool, focus groups shared the attitude that the platform itself was entertaining, offering collateral utility and benefits. Focus groups discussed different scenarios for using the metaverse. For example, a treatment for desire disorders could include exploring new forms of pleasure in the metaverse. However, users could also just use the virtual environment to experiment with sexual fantasies that they do not feel comfortable sharing with their real‐life partner. Although exploring sexual fantasies is not therapeutic in itself, it does represent an important part of sexual treatments [58]. Additionally, the repression of sexual fantasies reduces sexual well‐being [8, 11]. Therefore, even if it is not a psychological intervention per se, addressing this repression through the metaverse could help increase general sexual health. On a related note, there are no previous studies that analyse the frequency of people using metaverses for satisfying hidden sexual desires.

In addition to these facilitators to treatment, the focus groups held two negative attitudes, reservations or barriers about metaverse therapy and e‐mental health more broadly. Specifically, e‐mental health was viewed by the focus groups as less personal than in‐person therapy and more appropriate for younger people. These concerns are consistent with those described in previous literature [35]. The personal aspect is a challenge for all online therapy. It is noteworthy that one person among the total sample had previously received online therapy. She reported that although she initially had reservations about online therapy, she found out that there was no difference with traditional therapy. This suggests that previous experiences with e‐mental healthcare might allay concerns about the impersonal nature of metaverse therapy [28, 29, 30]. Previous studies have identified significant associations between perceived online therapeutic presence and attitudes towards online therapy [59]. More work needs to be done, though, regarding the relationship between prior experience with online psychological therapy and attitudes towards it.

The other main reservation among focus group participants regarding the use of metaverse therapy concerned age. Age differences in attitudes towards technology use have been found in previous studies [31, 32], and these differences were highlighted in the present study. Whereas younger people saw themselves as possible users of metaverse therapy, older people did not. It should be noted that although technology was seen as a barrier for older people, there was not a similar barrier for older people due to sex being a sensitive topic [60, 61]. In other words, the technology—not the topic—seemed to be what divided older and younger people in their reservations towards metaverse therapy. That said, experience with technology may change attitudes towards it. For example, the COVID‐19 pandemic forced many older people to use smartphones or computers to stay in touch with their loved ones. This experience may have changed their attitudes towards technology. Older people who feel more capable regarding technology are more likely to have better attitudes towards technology [62]. It is important, then, that healthcare providers implementing e‐mental health interventions also put effort into educating older patients to make them feel comfortable using the technology. Compared to the other focus groups, the older focus group used the internet less, which further suggests that technological literacy—rather than age—may be the ultimate reason for why older people have reservations using metaverse therapy. Although metaverses are still unknown to a wide range of people and education is needed for patients and professionals, the technology is growing and seems to be viewed favourably as a therapeutic tool.

In addition to the nine themes identified and discussed above, there are three additional findings of interest. First, individuals held similar attitudes regardless of whether or not they had received previous psychological treatment. This suggests that the acceptability of metaverse therapy may depend more on individual attitudes towards technology than attitudes towards therapy. Second, participants reported that they trusted a metaverse‐based treatment for mild and moderate disorders but not for severe mental illness. This seems to be consistent with the literature on app‐based intervention attitudes [34, 35]. Third, and finally, the trust in their therapist would be a crucial factor in the decision on using this kind of therapy or not. The trust in the therapist, commonly referred to as therapeutic alliance, has been found to be a key aspect to successful treatment [63]. This trust can be built in traditional therapy where metaverse therapy is used as a tool. For example, potential users would meet their psychologist in person while also performing specific tasks or conducting therapeutic modules inside a metaverse. However, trust could also be built inside the metaverse. Available literature suggests that it is possible to build trust in a therapist who a person has only met online [64]. Some authors [65] have started to collect data around this topic and have gathered some practical tips for assuring therapeutic alliance in e‐mental health interventions using videocalls [66]. It can help, for example, to set expectations and acknowledge the awkward parts of different modalities of psychotherapy (whether using a video call or the metaverse).

The attitudes identified in this study regarding e‐mental health and metaverse therapy warrant future research. The qualitative methodology here, ideal for exploratory purposes, has some limitations. To gather personal information rich in details, the sample size of three focus groups is relatively small. Future quantitative studies can use larger samples to tease out differences in group attitudes. Although there did not seem to be gender differences in this study, perhaps they would be revealed in a larger sample or if participants were divided by gender (if, e.g., men are more comfortable using technology or women are more comfortable with receiving therapy). Additionally, the sample did not include participants specifically diagnosed with sexual disorders. Given the high prevalence of sexual disorders among the general population [1], and the difficulty that individuals have in speaking about sexual issues due to stigma or shame [12, 14], it was decided to recruit the sample from the general population. However, future research is needed to be able to draw stronger conclusions regarding the barriers that people with sexual dysfunction face.

The present study leaves a number of other questions unanswered and ready for future research. How might technological experience change attitudes towards technology in general and online therapy in particular? What attitudinal differences exist regarding the various types of psychotherapy, the types of delivery and the role of the psychologist? To what extent is the therapeutic alliance or trust independent of the treatment delivery modality?

In conclusion, as it has been suggested with the treatment of depression or anxiety, for which e‐mental health interventions are well accepted [34], it seems that attitudes towards a metaverse‐based intervention for sexual problems are mainly positive. People perceive this new modality of therapy as an accessible, useful and interesting way to reach for help that, otherwise, they would not seek.

## Author Contributions

**Ariana Vila:** conceptualization (lead), methodology (lead), writing–original draft (lead), formal analysis (lead), writing–review and editing (equal). **Rosa Romero‐Moreno:** conceptualization (supporting), methodology (supporting), writing–review and editing (equal). **Celia Nogales‐Gonzalez:** conceptualization (supporting), methodology (supporting), writing–review and editing (equal). **Andrew J. Ritchey:** writing–review and editing (equal). **Juan Ardoy‐Cuadros:** conceptualization (supporting), methodology (supporting), writing–review and editing (equal).

## Conflicts of Interest

The authors declare no conflicts of interest.

## Supporting information

Supporting information.

## Data Availability

The data that support the findings of this study are available on request from the corresponding author. The data are not publicly available due to privacy or ethical restrictions.
