# Supplementary material for: Sexual Interventions in the Metaverse: Attitudes Towards Novel Therapeutic Approaches, a Qualitative Study
Source: Health Expect. 2024 Aug 29;27(5):e70004. doi: 10.1111/hex.70004 (PMC11360517; doi:10.1111/hex.70004)
Supplement: Supplementary file 1 — Supporting information. [file HEX-27-e70004-s001.docx]

**Supplementary material: Focus group script**

Good morning/afternoon everyone. My name is Ariana Vila, and I am the person conducting this study. Today, I am accompanied by [include names of those who will be present], who will not participate actively but are here solely to facilitate the technical aspects of the study, such as recording. First of all, I would like to thank you for your participation in the study and your presence here, and to discuss the session's development with you.

As you already know, we are trying to gain a deeper understanding of perceptions and attitudes towards conducting psychological treatments in virtual environments. Therefore, over the next two hours (approximately), I would like you to freely discuss your ideas and opinions on the subject after viewing the provided material. The goal is to replicate a conversation among yourselves about the topics that arise. There will be no exchange of questions and answers between you and us. You may address another participant without waiting for the moderator’s invitation.

It is important to note that there are no right or wrong comments or responses, but rather different perspectives on the same issue. I ask that you express your opinions freely, even if they may differ from those of another participant. Furthermore, if you disagree with something that has been mentioned, I would ask you to speak out so that all possible opinions of those present are heard. We are interested in both positive and negative comments in order to obtain the broadest perspective possible.

Also, as you were informed when signing the informed consent, the session will be recorded. So before we begin, you will need to confirm your consent to the recording. The content will only be accessible to the research team, and individuals will not be identifiable through their comments in future reports. Your anonymity will be preserved at all times. The recordings will be destroyed after 15 days.

If you agree, and if there are no further questions, we can begin...

1. Engagement questions.

- What is your name, and what things do you enjoy doing?
- Have you heard of Second Life before?
- Do you think it could be entertaining to join?
- Have you ever been to a psychologist?
- If you have been: What is your experience? If not: What is the reason? What would have supported you to go?

2. Exploratory questions.

- Advantages and disadvantages of using a metaverse-based therapy for mental health interventions/treating sexual disorders:
  - What advantages do you think this kind of therapy may have over traditional treatments?
  - What advantages do you think Second Life could have compared to traditional therapy?
  - What disadvantages do you see?
- Confidence/trust in metaverse-based therapy (strengths and weaknesses):
  - Would you use it?
  - For what reasons would you use it?
  - Would you recommend it to a friend who is struggling but doesn’t want to go to a psychologist?
- Problems/barriers they see:
  - Is there any reason why you would reject a metaverse-based therapy?
  - Is there any reason you wouldn't like to attend therapy in a virtual environment?

3. Exit questions.

- Is there anything else you would like to share or say?
